# Associations of Coffee, Diet Drinks, and Non-Nutritive Sweetener Use with Depression among Populations in Eastern Canada

**DOI:** 10.1038/s41598-017-06529-w

**Published:** 2017-07-24

**Authors:** Zhijie M. Yu, Louise Parker, Trevor J. B. Dummer

**Affiliations:** 1 Population Cancer Research Program and Department of Pediatrics, Dalhousie University, Halifax, NS B3H 4R2 Canada; 2 Centre of Excellence in Cancer Prevention, School of Population and Public Health, University of British Columbia, Vancouver, BC V6T 1Z3 Canada

## Abstract

Consumption of coffee and diet drinks and the use of non-nutritive sweeteners is commonplace worldwide. We conducted a cross-sectional analysis to investigate the associations between coffee consumption and non-nutritive sweetener use and depression among populations in Atlantic Canada. During 2009 to 2013, we recruited 18838 participants aged 35–69 years (5854 men and 12984 women) for the baseline survey of the Atlantic Partnership for Tomorrow’s Health cohort study. Coffee consumption, sweetener use, and major depression were assessed using a set of standardized questionnaires. We utilized multiple logistic regression models to assess the associations of coffee drinking and non-nutritive sweetener use with major depression. Compared with non-coffee drinkers, female participants who drank coffee ≥4 cups/day had an odds ratio of 1.38 (95% confidence interval, 1.15–1.64) for major depression with adjustment for sociodemographic and behavioral factors, chronic disease status, and body mass index. We found a significant association between depression and consumption of sweeteners and diet drinks, which was more apparent among women than men. We conclude that heavy coffee drinking and non-nutritive sweetener use were associated with depression among populations in Atlantic Canada. Further studies are warranted to investigate the underlying biological mechanisms.

## Introduction

Depression is an increasing public health concern that influences both quality of life and disease outcome^1^. Risk factors associated with depression are not fully understood. Coffee is the second most popular source of beverage intake in Canada, accounting for around 80% of daily caffeine intake among Canadian adults^2^. Non-nutritive sweeteners (NNS) have been included in the Canadian nutritional guidelines for decades in terms of cardiometabolic disease control and prevention^3^. A national survey showed that Canadians consume large quantities of diet drinks^2^. Few studies have assessed whether depression, coffee drinking, and NNS use are correlated among Canadians.

Some evidence indicates that those who drink coffee, compared with non-coffee drinkers, have a decreased risk of developing type 2 diabetes, coronary heart disease, and stroke^4^. However, the relationship between heavy coffee consumption and overall health remains controversial. Recently, two large-scale population-based longitudinal studies reported contrasting associations between heavy coffee drinking (≥4 cups/day) and all-cause mortality^5, 6^. There are also some inconsistencies apparent in meta-analysis studies. Two meta-analyses showed that coffee consumption was inversely associated with cardiovascular disease (CVD) and all-cause mortality^7, 8^. One meta-analysis found that moderate coffee drinking was inversely associated with CVD mortality, with the lowest risk being observed at three to five cups per day. Heavy coffee drinking (>5 cups/day) was not related to decreased CVD mortality^9^. Further, a large-scale observational and Mendelian randomization study reported a U-shaped association between coffee consumption and CVD and all-cause mortality. However, genetic variations that influence caffeine intake was not related to either CVD or all-cause mortality^10^. Similarly, the epidemiologic evidence to date concerning coffee consumption and depression has been contradictory; studies have reported both increased and decreased risk of depression with coffee drinking^11–14^, though two recent systematic reviews suggested an inverse association^15, 16^. These findings may imply that other factors that are associated with coffee consumption may play a role in the observed associations between coffee drinking and health outcomes.

Regarding the health consequences of NNS use, a recent study from the US reported an association between consumption of diet drinks and depression^17^. Two systematic reviews raised a concern regarding the effects of diet drink consumption on body weight control^18^ and type 2 diabetes prevention^19^. As consumption of coffee and diet drinks and the use of artificial sweeteners is commonplace in Canada^2, 3^, we hypothesized that coffee drinking, diet drink consumption, and NNS use might be correlated and related to depression among Canadians. Therefore, we assessed the associations of coffee drinking, diet drink consumption, and NNS use with depression amongst people aged 35 to 69 years old participating in the baseline survey of the Atlantic Partnership for Tomorrow’s Health (Atlantic PATH) cohort study.

## Results

### General characteristics of study participants

Among the study participants, 62.4% reported coffee drinking. Of these, 52.7% drank regular coffee, 5.7% drank decaffeinated coffee, and 4.0% drank both. For NNS use, 44.3% reported the consumption of either NNS or diet drinks. Of these, 5.3% added NNS to coffee or tea, 31.9% drank diet drinks, and 7.1% reported both. The overall prevalence of major depression was 17.1% and was significantly higher in women than men (19.7% versus 11.2%, *P* < 0.001, Table 1 and Supplemental Tables 1 and 2). Coffee drinking and NNS use were significantly correlated, and they were also positively correlated with depressive symptom scores (Table 2).

**Table 1:** General characteristics of study participants by gender*.

Characteristics	Men (n = 5854)		Women (n = 12984)		*P* value
**Age, yr**	53.8	(9.2)	52.5	(8.9)	<0.001
**Regular coffee, n (%)**					
Never	2107	(36.0)	6053	(46.6)	
>0–<4 cups/day	3011	(51.4)	6229	(48.0)	<0.001
≥4 cups/day	736	(12.6)	702	(5.4)	
**Decaffeinated coffee, n (%)**					
Never	5406	(92.4)	11598	(89.3)	
>0–<2 cups/day	231	(4.0)	866	(6.7)	<0.001
≥2 cups/day	217	(3.7)	520	(5.0)	
**Total coffee, n (%)**					
Never	1838	(31.4)	5243	(40.4)	
>0–<4 cups/day	3196	(54.6)	6856	(52.8)	<0.001
≥4 cups/day	820	(14.0)	885	(6.8)	
**NNS in coffee or tea, n (%)**					
Never	5195	(88.7)	11306	(87.1)	
Some-times	190	(3.3)	567	(4.4)	0.27
Always	469	(8.0)	1111	(8.6)	
**Diet drinks, n (%)**					
Never	3709	(63.4)	7788	(60.0)	
<1 time/week	1198	(20.5)	3228	(24.9)	<0.001
1–6 times/week	604	(10.3)	1324	(10.2)	
≥1 times/day	343	(5.9)	644	(5.0)	
**NNS in coffee, tea, or drinks, n (%)**					
Never	3416	(58.4)	7077	(54.5)	
Either	2072	(35.4)	4940	(38.1)	0.16
Both	366	(6.3)	967	(7.5)	
**Major depression** ^†^, **n (%)**	656	(11.2)	2561	(19.7)	<0.001
**Province of residence, n (%)**					
Nova Scotia	3654	(64.3)	8091	(62.3)	0.24
New Brunswick	1354	(23.1)	3089	(23.8)	
Newfoundland and Labrador	746	(12.7)	1550	(11.9)	
Prince Edward Island	100	(1.7)	254	(2.0)	
**Ethnicity, n (%)**					
White	4997	(85.4)	10861	(83.7)	
Non-white	293	(5.0)	749	(5.8)	0.01
DNK/PNA^c^	564	(9.6)	1374	(10.6)	
**Education, n (%)**					
Less than high school	1145	(19.6)	2247	(17.3)	
College level	2073	(35.4)	5213	(40.2)	<0.001
University level or higher	2357	(40.3)	4929	(38.0)	
DNK/PNA^c^	279	(4.8)	595	(4.6)	
**Smoking status, n (%)**					
Never smoker	2641	(45.1)	6485	(50.0)	
Former smoker	2344	(40.0)	4701	(36.2)	<0.001
Current smoker	582	(9.9)	1141	(8.8)	
DNK/PNA^c^	287	(4.9)	657	(5.1)	
**Alcohol use, n (%)**					
Abstainer	595	(10.2)	297	(10.4)	
Occasional drinker	1637	(28.0)	1463	(44.6)	
Regular drinker	2068	(35.3)	953	(27.7)	<0.001
Habitual drinker	1262	(21.6)	456	(12.7)	
DNK/PNA^c^	292	(5.0)	616	(4.7)	
**Cardiovascular disease** ^‡^ **, n (%)**	313	(5.4)	273	(2.1)	<0.001
**Diabetes mellitus** ^§^ **, n (%)**	459	(7.8)	970	(7.5)	0.37
**Body weight, kg**	89.6	(15.0)	74.4	(14.4)	<0.001
**Body height, cm**	175.8	(6.6)	162.3	(6.0)	<0.001
**Body mass index, kg/m** ^**2**^	28.9	(4.5)	28.1	(6.0)	<0.001
**Total physical activity, MET-min/week**	2799	(1156, 6294)	1980	(787, 4305)	<0.001
**Healthy eating index**	35.3	(11.1)	38.9	(11.4)	<0.001

DNK/PNA = do not know/prefer not to answer; NNS = non-nutritive sweetener. ^*^Data are means (standard deviation), number of participants (percentage), or median (interquartile range). Percentages may not total 100 due to rounding. The numbers of participants vary slightly among different variables. ^†^Defined as a PHQ-9 score ≥ 10 and/or current use of antidepressant. ^‡^Self-reported coronary heart disease and/or stroke. ^§^Self-reported diabetes mellitus.

**Table 2:** Spearman partial correlation coefficients between depressive symptom score, coffee drinking, and non-nutritive sweetener use among study particiapnts^*^.

	Depressive symptom score	Total coffee	Regular coffee	Decaffeinated coffee	Any NNS use	NNS in coffee or tea
**Total coffee**	0.07^b^	—				
**Regular coffee**	0.07^b^	0.91^b^	—			
**Decaffeinated coffee**	0.02^a^	0.25^b^	−0.11^b^	—		
**Any NNS use**	0.10^b^	0.11^b^	0.09^b^	0.06^b^	—	
**NNS use in coffee or tea**	0.07^b^	0.11^b^	0.09^b^	0.05^b^	0.55^b^	—
**Diet drinks**	0.08^b^	0.06^b^	0.04^b^	0.04^b^	0.87^b^	0.16^b^

NNS = non-nutritive sweetener. *Adjusted for age and sex. ^a^ *P* < 0.05, ^b^ *P* < 0.001.

Compared with women, men were more likely to drink regular coffee and be a heavy coffee drinker (≥4 cups/day) but less likely to consume diet drinks (Table 1). Moreover, compared with non-coffee drinkers, heavy coffee drinkers were more likely to be highly educated, be current smokers and alcohol drinkers, and have cardiovascular disease. Meanwhile, they also had higher levels of BMI, physical activity, and diet quality (Supplemental Table 1). Compared with non-NNS users, participants who both added NNS in coffee or tea and consumed diet drinks were less likely to be highly educated and were more likely to have cardiovascular disease and diabetes. They also had higher levels of BMI, physical activity, and diet quality (Supplemental Table 2).

### Associations between coffee drinking and major depression

Compared with non-coffee drinkers, female participants who drank ≥4 cups/day of any form of coffee had an increased likelihood of having major depression (odds ratio [OR], 1.46; 95% confidence interval [CI], 1.24–1.73) with adjustment for age (Table 3). After further controlling for sociodemographic and lifestyle factors, health status, and BMI, the association between heavy coffee consumption and increased depression remained statistically significant (OR, 1.38; 95% CI, 1.15–1.64). The stratified analyses by two types of coffee showed that this association was apparent only in regular coffee drinkers.

**Table 3:** Associations between coffee consumption and major depression risk among populations in Atlantic Canada.

	Cases/n	OR (95% CI)	
		Model 1	Model 2
***Regular coffee***			
*Men*			
Never	241/2107	1.0 (Reference)	1.0 (Reference)
>0–<4 cups/day	314/3011	0.90 (0.75–1.07)	0.93 (0.77–1.14)
≥4 cups/day	101/736	1.23 (0.95–1.57)	1.11 (0.84–1.45)
***P*** **for trend**		0.42	0.69
*Women*			
Never	1198/6053	1.0 (Reference)	1.0 (Reference)
> 0–<4 cups/day	1174/6229	0.94 (0.86–1.03)	1.02 (0.92–1.12)
≥4 cups/day	189/702	1.49 (1.25–1.78)	1.35 (1.11–1.63)
***P*** **for trend**		0.097	0.036
***P*** **for interaction between men and women**		0.45	0.55
***Decaffeinated coffee***			
*Men*			
Never	603/5406	1.0 (Reference)	1.0 (Reference)
> 0–<2 cups/day	23/231	0.89 (0.57–1.38)	1.04 (0.66–1.64)
≥2 cups/day	30/217	1.29 (0.87–1.92)	1.11 (0.73–1.70)
***P*** **for trend**		0.37	0.60
*Women*			
Never	2284/11598	1.0 (Reference)	1.0 (Reference)
> 0–<2 cups/day	161/866	0.94 (0.79–1.12)	1.02 (0.85–1.22)
≥2 cups/day	116/520	1.19 (0.96–1.47)	1.18 (0.95–1.47)
***P*** **for trend**		0.31	0.19
***P*** **for interaction between men and women**		0.90	0.999
***Total coffee***			
*Men*			
Never	213/1838	1.0 (Reference)	1.0 (Reference)
>0–<4 cups/day	327/3196	0.87 (0.72–1.04)	0.90 (0.73–1.11)
≥4 cups/day	116/820	1.25 (0.98–1.60)	1.11 (0.85–1.45)
***P*** **for trend**		0.29	0.65
*Women*			
Never	1035/5243	1.0(Reference)	1.0 (Reference)
>0–<4 cups/day	1292/6856	0.94 (0.86–1.03)	1.03 (0.93–1.13)
≥4 cups/day	234/885	1.46 (1.24–1.73)	1.38 (1.15–1.64)
***P*** **for trend**		0.030	0.009
***P*** **for interaction between men and women**		0.54	0.42

Model 1, adjusted for age. Model 2, further adjusted for ethnicity, education, province of residence, smoking status, alcohol drinking, self-reported cardiovascular disease and diabetes, healthy eating index (in tertiles), total physical activity (in MET-min/week tertiles), and BMI based on model 1.

### Associations between sweetener use and major depression

In analyses adjusted only for age, daily addition of NNS in coffee or tea and consumption of diet drinks were associated with a significantly increased likelihood of having major depression in both men and women (Table 4). After controlling for the covariables, the association was more apparent amongst women than men. In the multivariable adjusted logistic regression analyses, compared with those who never added NNS in coffee or tea, both male and female participants who always added NNS in coffee or tea had significantly increased likelihoods for major depression (OR, 1.39; 95% CI, 1.05–1.83 for males and OR, 1.31; 95% CI, 1.13–1.53 for females). For diet drinks, the association was apparent among women only. Compared with those who never drank diet drinks, female participants who drank diet drinks ≥1 times/day had an increased likelihood for major depression (OR, 1.66; 95% CI, 1.38–2.00). In the combined analyses, female participants who both added NNS in coffee or tea and drank diet drinks had an increased likelihood for major depression (OR, 1.32; 95% CI, 1.12–1.55) compared with those who did not add NNS in coffee or tea or drink diet drinks.

**Table 4:** Associations between non-nutritive sweeteners use and major depression risk among populations in Atlantic Canada.

	Cases/n	OR (95% CI)	
		Model 1	Model 2
***NNS in coffee or tea***			
*Men*			
Never	543/5195	1.0 (Reference)	1.0 (Reference)
Sometimes	30/190	1.60 (1.08–2.39)	1.29 (0.85–1.95)
Always	83/469	1.85 (1.43–2.38)	1.39 (1.05–1.83)
***P*** **for trend**		<0.001	0.013
*Women*			
Never	2133/11306	1.0 (Reference)	1.0 (Reference)
Sometimes	127/567	1.24 (1.01–1.52)	1.14 (0.92–1.41)
Always	301/1111	1.59 (1.38–1.83)	1.31 (1.13–1.53)
***P*** **for trend**		<0.001	<0.001
***P*** **for interaction between men and women**		0.34	0.39
***Diet drinks***			
*Men*			
Never	394/3709	1.0 (Reference)	1.0 (Reference)
<1 time/week	124/1198	0.97 (0.79–1.20)	0.96 (0.77–1.21)
1–6 times/week	73/604	1.16 (0.89–1.52)	1.04 (0.78–1.38)
≥1 times/day	65/343	1.97 (1.48–2.64)	1.35 (0.98–1.86)
***P*** **for trend**		<0.001	0.17
*Women*			
Never	1467/7788	1.0 (Reference)	1.0 (Reference)
<1 time/week	599/3228	0.98 (0.88–1.09)	0.95 (0.85–1.06)
1–6 times/week	275/1324	1.13 (0.98–1.30)	1.03 (0.89–1.20)
≥1 times/day	220/644	2.22 (1.87–2.64)	1.66 (1.38–2.00)
***P*** **for trend**		<0.001	<0.001
***P*** **for interaction between men and women**		0.91	0.97
***NNS in coffee, tea, or drinks***			
*Men*			
Never	341/3416	1.0 (Reference)	1.0 (Reference)
Either^*^	255/2072	1.27 (1.07–1.51)	1.17 (0.97–1.41)
Both^†^	60/366	1.78 (1.32–2.39)	1.23 (0.89–1.71)
***P*** **for trend**		<0.001	0.077
*Women*			
Never	1292/7077	1.0 (Reference)	1.0 (Reference)
Either^*^	1016/4940	1.16 (1.06–1.27)	1.05 (0.95–1.15)
Both^†^	253/967	1.58 (1.35–1.84)	1.32 (1.12–1.55)
***P*** **for trend**		<0.001	0.006
***P*** **for interaction between men and women**		0.58	0.34

NNS = non-nutritive sweetener. Model 1, adjusted for age. Model 2, further adjusted for ethnicity, education, province of residence, smoking status, alcohol drinking, self-reported cardiovascular disease and diabetes, healthy eating index (in tertiles), total physical activity (in MET-min/week tertiles), and BMI based on model 1. ^*^Either added non-nutritive sweetener in coffee or tea or drank diet drinks. ^†^Both added non-nutritive sweetener in coffee or tea and drank diet drinks.

In the both sexes combined analyses, both heavy coffee drinking and any NNS use were associated with increased likelihoods of having major depression after adjustment for all the covariables (Table 5). In the stratified analysis by two major depression risk factors (Table 6), heavy coffee drinking was associated with major depression across all smoking and overweight or obesity strata. Whereas, the association between NNS use and major depression was apparent only among those who were never smokers and with overweight or obesity. In the smoking and BMI combined analyses, the associations were evident among those who were never smokers with normal weight and those who were ever smokers with overweight or obesity.

**Table 5:** Interactions of coffee drinking and sweeteners use in relation to major depression risk^*^.

Total coffee drinking	Use of NNS	
	Never	Any^†^
Never	1.0 (Reference)	1.20 (1.05–1.38)
>0–<4 cups/day	1.04 (0.92–1.18)	1.13 (1.00–1.28)
≥4 cups/day	1.44 (1.18–1.75)	1.39 (1.13–1.70)

NNS = non-nutritive sweetener. *ORs and 95% CIs were computed with adjustment for age, sex, ethnicity, education, province of residence, smoking status, alcohol drinking, self-reported cardiovascular disease and diabetes, healthy eating index (in tertiles), total physical activity (in MET-min/week tertiles), and BMI. ^†^Non-nutritive sweetener use in coffee or tea or diet drinks.

**Table 6:** Multivariable adjusted odds ratios (95% confidence intervals) for depression according to total coffee drinking and non-nutritive sweeteners use among participants with different smoking status and BMI levels (n = 17894)*.

	Smoking^†^		Overweight or obesity^‡^		Combined		
	Never smoker	Ever smoker	No	Yes	Never smoker with normal weight	Ever smoker or overweight or obesity	Ever smoker with overweight or obesity
**Total coffee drinking**							
Never	1.0 (Reference)	1.0 (Reference)	1.0 (Reference)	1.0 (Reference)	1.0 (Reference)	1.0 (Reference)	1.0 (Reference)
>0–<4 cups/day	1.07 (0.94–1.21)	0.89 (0.78–1.01)	1.08 (0.90–1.30)	0.93 (0.84–1.04)	1.15 (0.90–1.48)	1.04 (0.91–1.18)	0.86 (0.74–0.99)
≥4 cups/day	1.29 (1.00–1.67)	1.27 (1.06–1.52)	1.49 (1.09–2.02)	1.21 (1.03–1.43)	1.84 (1.16–2.91)	1.25 (0.98–1.59)	1.26 (1.03–1.54)
*P* for trend	0.066	0.13	0.032	0.30	0.023	0.14	0.21
**Use of NNS**							
Never	1.0 (Reference)	1.0 (Reference)	1.0 (Reference)	1.0 (Reference)	1.0 (Reference)	1.0 (Reference)	1.0 (Reference)
Any^§^	1.13 (1.00–1.28)	1.07 (0.96–1.20)	1.04 (0.88–1.24)	1.13 (1.03–1.24)	1.36 (1.07–1.73)	0.97 (0.86–1.10)	1.17 (1.03–1.33)
*P*	0.055	0.36	0.64	0.021	0.011	0.63	0.63

NNS = non-nutritive sweetener. *Adjusted for age, sex, ethnicity, education, province of residence, alcohol drinking, smoking (where applicable), self-reported cardiovascular disease and diabetes, healthy eating index (in tertiles), total physical activity (in MET-min/week tertiles), and BMI as well as mutually controlled for coffee drinking and sweeteners use. ^†^Smoking status: never/ever smoker. ^‡^Overweight or obesity: BMI ≥ 25 kg/m^2^. ^§^Non-nutritive sweetener use in coffee or tea or diet drinks.

## Discussion

In this large-scale, population-based cross-sectional analysis, we found that coffee drinking and NNS use were popular among participant populations of the Atlantic PATH study. Depression, coffee drinking, and NNS use were positively correlated. Heavy coffee drinking and NNS use were associated with major depression. This association was independent of sociodemographic and behavioral factors, chronic disease status, and body weight, but was more evident among women than men. In addition, the associations of heavy coffee drinking and NNS use with depression appeared to be independent of smoking and obesity, which are known risk factors for major depression.

Our data showed that heavy coffee drinkers and NNS users were more likely to have a worse risk factor profile for cardiometabolic disease compared with non-coffee drinkers and non-NNS users. The phenomenon has been observed in some longitudinal studies assessing the associations of coffee drinking with the risk of all-cause mortality^5, 6, 20^. The most consistent trend among the three large-scale population-based studies is that heavy coffee drinkers (≥4 cups/day) had significantly higher prevalence of smoking compared with non-coffee drinkers. Two out of the three studies reported that heavy coffee drinkers had either relatively higher levels of BMI^5^ or prevalence of overweight or obesity^20^ than non-coffee drinkers. Interestingly, heavy coffee drinking was associated with increased risk of all-cause mortality in the simple adjusted models among all the three studies^5, 6, 20^. After multivariable adjustment, two studies showed an inverse association between heavy coffee drinking and all-cause mortality^6, 20^. Whereas one positive association remained^5^. Further stratified analyses revealed that the multivariable adjusted inverse association between heavy coffee drinking and all-cause mortality existed only among those non-smokers but not current smokers^6, 20^. These observations might imply that the association between heavy coffee drinking and all-cause mortality might be affected by risk factor profiles of the heavy coffee drinkers who participated in these studies.

Our results are consistent with data from some studies^11, 12, 21^ but not others^13, 22, 23^ that reported associations between coffee drinking and mental health. Among these studies, Lucas *et al*. reported an inverse association between coffee drinking and risk of developing depression in the Nurses’ Health Study^22^. In this study, women who reported coffee drinking ≥4 cups/day had lower non-smoking rate compared with those who drank ≤1 cup/week, and there was no association between coffee drinking and depression risk in the age-adjusted analysis. The inverse association appeared after multivariable adjustment for major lifestyle risk factors and BMI.

In our cross-sectional analyses, we found that the positive association between heavy coffee drinking and depression was evident among both low and high risk individuals in terms of smoking and BMI levels. As coffee drinking is the primary source of caffeine intake among Canadians^2^, it is likely that higher coffee consumption may result in the higher intake of caffeine. Depressed individuals might choose to consume more caffeine, for example as a form of self-medication^24^.

In addition to individual studies, results from meta-analyses have also been inconsistent. A U-shaped association between coffee drinking and CVD mortality was reported in one study^9^ but not replicated by two other studies^7, 8^. Two meta-analyses showed that coffee consumption was consistently and inversely associated with depression, although this trend was not apparent for caffeine intake^15, 16^. Regarding other cognitive disorders, one study reported that the protective effect of coffee consumption on Parkinson’s disease risk was found at 3 cups/day^25^. By contrast, Kim *et al*. reported that there were no associations detected between coffee consumption and cognitive disorders^26^. In another study, pooled analyses did not show an association between habitual coffee consumption and risk of cognitive decline or dementia, but in sub-group analyses, there was an inverse association between coffee consumption and Alzheimer disease^27^. However, a major concern is that these pooled analyses rarely controlled for confounding factors across the selected studies, which also makes direct comparison with our findings problematic. Indeed, inconsistencies in findings across different studies suggests that unmeasured confounding is an important issue. A Mendelian randomization study design has a unique advantage in this respect because genetic variations in the targeted exposure are not related to the confounders^28^. By using this approach, a Danish study found that the genetic variations that are significantly related to caffeine intake were not associated with mortality from CVD and all-causes^10^. These findings imply that a Mendelian randomization study design may help elucidate whether the observed associations between coffee consumption and mental health is due to the effects of residual confounding factors or the effects of caffeine intake per se. In this regard, further well-designed studies, such as Mendelian randomization studies, are needed to confirm our findings and investigate the possible underlying biological mechanisms.

Though NNS use in foods is increasing, particularly among developed countries, data on the health consequences of NNS use are scarce. More recently, a study found a positive association between NNS use and depression risk among a large sample of older US adults^17^. We found that NNS users were more likely to report having diabetes in our study participants. Some studies reported that people with diabetes mellitus are associated with increased risk for depression^29^. However, we cannot rule out, due to the cross-sectional nature of the study, that the observed association we found is a result of reverse causality. Obese individuals have also been shown to be more likely to consume beverages and foods containing NNS^30^. Meanwhile, obese individuals are more likely to develop major depression^31^. Mattes and Popkin^32^ reported that the increasing use of NNS in foods parallels the increase in BMI in populations from industrialized countries. There is currently no direct evidence linking NNS use with obesity risk, but a Canadian study observed that daily NNS use among pregnant women was associated with increased infant BMI compared with the counterparts of non-NNS users^33^. Among our study participants, about a half reported the consumption of either NNS or diet drinks, and our data suggested that individuals who reported the consumption of NNS or diet drinks might have more risk factors associated with depression compared with those who did not. The association between NNS use and depression was apparent only among those who were overweight or obese. Given the high average levels of BMI in both men and women and the close relationship between obesity and depression, further studies are needed to investigate whether the interactions between heavy coffee drinking and NNS use may influence depression development.

Participants who were heavy coffee drinkers or frequent NNS users had relatively higher levels of BMI and lower levels of educational attainment. Meanwhile, they were more likely to be a current smoker and have cardiometabolic diseases. Most of these factors have been proven to be associated with the increased depression risk and are modifiable^34^. Our findings might imply that appropriate interventional programs tailored for cardiometabolic risk factor modifications targeting people who are heavy coffee drinkers and frequent NNS users may help ameliorate depressive symptoms and related comorbidities among participant populations.

The large sample size and well-controlled confounding factors are major advantages of this study. We also plotted depression risk factors by different levels of coffee drinking and frequencies of NNS use. Nonetheless, our analysis has some limitations. The majority of our study participants are white, and participants were not randomly selected from the populations in the Atlantic Canada provinces, this may limit the generalizability of our study findings to other populations. However, this is not necessarily a shortcoming for examining the association of depressive symptom with beverage consumption. In our analyses, we defined major depression with a set of validated questionnaires^35^ but did not include clinical diagnosis by a physician. This method might lead to some miss-classifications of the study outcomes and consequently result in an underestimation of the observed associations. We assessed NNS use as frequencies of daily intake but not the amount; this might compromise the strength of the observed associations as well. The associations of heavy coffee drinking and NNS use with depression were more apparent among women than men. This finding might be due to that the sample size of male participants was smaller than females and men had significantly lower prevalence of depression than women. The *P* values for interactions between men and women were not statistically significant indicating that men and women had similar trends in these associations. Finally, the cross-sectional nature of the study means we cannot infer causation from these results.

In summary, our findings suggested that depressive symptoms, coffee drinking, and NNS use were correlated among populations in Atlantic Canada. Participants who reported heavy coffee drinking and NNS use had increased likelihoods for major depression compared with those who never drank coffee or consumed NNS. Meanwhile, these participants also had more risk factors associated with depression development. These findings may suggest that further research is needed to elucidate how coffee and NNS influence depression development among populations in Atlantic Canada and the underlying biological mechanisms.

## Methods

### Study population

The Atlantic PATH is part of the Canadian Partnership for Tomorrow Project (CPTP), a pan-Canadian longitudinal cohort study examining the role of genetic, environmental, behavioral and lifestyle factors in the development of cancer and chronic disease^36^. The Atlantic PATH study is a population-based cohort in which study participants were residents of one of the four Atlantic Canada Provinces (Nova Scotia, New Brunswick, Newfoundland and Labrador, and Prince Edward Island) and aged between 35 and 69 years. Study participants were recruited from the general public through a combination of community outreach and awareness activities, advertising and media campaigns, and invitation. The 18838 Atlantic PATH study participants (5854 men and 12984 women) included in this analysis were recruited from the four Atlantic Canada provinces between 2009 and 2013. The study was approved by appropriate Research Ethics Boards (REB) in each Province, including Nova Scotia Health Authority REB, Vitalite Health Network REB, Horizon Health Network REB, Newfoundland and Labrador Health Research REB, University of Prince Edward Island REB, and Health PEI REB. All individuals provided written informed consent prior to participation. All data collection procedures followed the research protocol guidelines as approved by the Research Ethics Boards.

### Data collection

Data collection has been reported previously^37, 38^. A set of standardized questionnaires on sociodemographic, health, diet, and behavioral factors and self-measured anthropometric indexes were completed by the participants, and physical measurements (including anthropometric indexes and body composition) were measured by research nurses at an assessment center.

### Assessment of coffee drinking and use of non-nutritive sweeteners

Study participants answered questions on the number of standard sized cups of regular or decaffeinated coffee consumed per day. Eight ounces (250 milliliters) is the standard coffee cup size in Canada^2^. We grouped coffee drinking as: never, >0–< 4 cups/day, and ≥4 cups/day, and decaffeinated coffee drinking as: never, >0–< 2 cup/day, and ≥2 cups/day. Total coffee drinking was grouped as: never, >0–< 4 cups/day, and ≥4 cups/day.

NNS use was assessed based on participants’ responses to questions on the frequencies of adding NNS to coffee or tea and the consumption of diet drinks. NNS use was classified as never, sometimes, and always. Diet drinks are sugar-free and artificially sweetened beverages without calories^2^. Frequencies of diet drink consumption were classified as never, <1 time/week, 1–6 times/week, and ≥1 times/day. To count for both NNS use and diet drink consumption, we classified participants as never users for those who never used NNS or drank diet drinks, either users for those who reported either use of NNS or consumption of diet drinks, and both users for those who had positive responses to questions on both use of NNS and consumption of diet or drinks.

### Assessment of depressive symptoms and antidepressant use

We used the Patient Health Questionnaire (PHQ-9) to calculate a depression severity score for study participants^35^. The questionnaire includes nine items. Based on participants’ responses to the nine questions, a total score ranging from 0 (minimum) to 27 (maximum) was summarized for each individual. Self-reported current medication use was collected; all medications were coded according to the Nova Scotia Formulary (NSF) (www.nspharmacare.ca)^39^. The ATC Code N06A (antidepressants) was used to define antidepressant use. Major depression was defined as a PHQ-9 score ≥10 and/or current use of antidepressants^35, 37^.

### Assessment of covariables

Body weight was measured using the Tanita bioelectrical impedance device (Tanita BC-418, Tanita Corporation of America Inc., Arlington Heights, Illinois)^37^. Height was measured with a Seca stadiometer. Among the 18838 study participants, 13581 participants undertook body weight measurements, and 13635 participants undertook body height measurements, respectively. There were 10048 participants reported self-measured body weight and 10057 participants reported self-measured body height, respectively. Totally, 13579 participants had both measured weight and height and 5259 participants had self-reported anthropometric measures. For self-reported body weight, height, and body mass index (BMI), we made sex-specific corrections derived from the measured data^40^. BMI was calculated as weight in kilograms divided by height in meters squared.

Dietary intake for each study participant was derived from a semi-quantitative food frequency questionnaire^37, 38^. A healthy eating index (HEI)^37^ was developed according to the methods recommended for Americans and Canadians regarding food quality and prevention of chronic disease. The HEI is a continuous variable with a perfect score of 60 and a minimum score of 0. The higher the score, the healthier the diet. HEI scores were categorized as sex-specific tertiles in the multivariable regression analyses^37^.

We assessed physical activity using the International Physical Activity Questionnaire (IPAQ), long form version^41^. Metabolic equivalent (MET) minutes per week were calculated according to the IPAQ scoring protocol^42^. Levels of total physical activity were categorized as low, medium, and high by the sex-specific total metabolic expenditure (MET-min/week) tertiles.

Ethnicity was grouped as white and non-white. Educational attainment was categorized as high school or lower, college level, and university level or higher. For smoking behavior, participants were grouped as never smoker, former smoker, and current smoker. For alcohol drinking, respondents were classified as abstainer, occasional drinker, regular drinker, and habitual drinker^37^. Self-reported chronic disease was dichotomized as yes or no for cardiovascular disease (coronary heart disease or stroke) and diabetes mellitus.

### Statistical analyses

Differences in study participant characteristics between men and women and across total coffee consumption and NNS use were tested with the *Student-t* test for continuous variables, except for physical activity which was examined with the Wilcoxon rank sum test, and the *chi-square* test for categorical variables. We computed Spearman partial correlation coefficients between depressive symptom score, coffee drinking, and NNS use with adjustment for age and sex.

Multivariable logistic regression was used to evaluate the associations of coffee drinking and NNS use with major depression. In the multivariable regression analyses, we controlled for sociodemographic and behavioral factors, chronic disease, and BMI. We computed *P* values for trend by treating variables of coffee drinking and NNS use as the continuous variable and *P* values for interaction between men and women by including the interaction terms of sex and coffee drinking or NNS use in the models. Moreover, we explored the possible interactions between total coffee consumption and NNS use in relation to risk of major depression with adjustment for all of the covariables. In addition, we did a stratified analysis among those with non-missing values in both smoking and BMI (n = 17894), as we previously found that smoking and obesity were associated with major depression^37^. In the stratified analysis, participants were classified as never smoker or ever smoker and normal weight or overweight or obesity (BMI ≥ 25 kg/m^2^). ORs and 95% CIs were computed for major depression according to coffee drinking and NNS use in each stratum with appropriate adjustment for the covariables. Statistical significance was defined as *P* < 0.05 (two-sided). Data management and analyses were performed with SAS for Windows, version 9.4 (SAS Institute, Cary, North Carolina).

### Data availability statement

No datasets were generated during the current study. Data from the Atlantic PATH study is not publically available to researchers without an approved data access request to Atlantic PATH, as per the Atlantic PATH consent form and study protocol. While the authors cannot provide data to a third party directly, the dataset can be provided by Atlantic PATH following a data access request approval. Interested researchers should contact Atlantic PATH (http://atlanticpath.ca/).

### Disclosure statement

This study was partly presented at the 2013 Canadian Society for Epidemiology and Biostatistics Biennial Conference, St. John’s, Newfoundland, June 24–27, 2013.

## Electronic supplementary material

Supplementary Information
