# Supplementary material for: Associations of Coffee, Diet Drinks, and Non-Nutritive Sweetener Use with Depression among Populations in Eastern Canada
Source: Sci Rep. 2017 Jul 24;7:6255. doi: 10.1038/s41598-017-06529-w (PMC5524745; doi:10.1038/s41598-017-06529-w)
Supplement: Supplementary file 1 — Supplementary Information [file 41598_2017_6529_MOESM1_ESM.pdf]

Associations of Coffee, Diet Drinks, and Non-Nutritive Sweetener Use with Depression among Populations in Eastern Canada

Zhijie M Yu[1], Louise Parker[1], Trevor J B Dummer[2]

[1] Population Cancer Research Program and Department of Pediatrics, Dalhousie University, Halifax, NS, Canada, B3H 4R2

[2] Centre of Excellence in Cancer Prevention, School of Prevention and Public Health, University of British Columbia, Vancouver, BC, Canada, V6T 1Z3

Supplemental Table 1. Characteristics of participants according to total coffee consumption[*]

Characteristics	Total coffee consumption, cups/day						*P* value
	**Never** (n = 7081)		**> 0-< 4** (n = 10052)		**≥ 4** (n = 1705)		
**Age, yr**	52.8	(9.1)	52.9	(9.0)	52.8	(8.7)	0.75
**Sex, n (%)**							
Men	1838	(26.0)	3196	(31.8)	820	(48.1)	<0.001
Women	5243	(74.0)	6856	(68.2)	885	(51.9)	
**NNS in coffee or tea, n (%)**							
Never	6594	(93.1)	8441	(84.0)	1466	(86.0)	
Some-times	150	(2.1)	538	(5.4)	69	(4.1)	<0.001
Always	337	(4.8)	1073	(10.7)	170	(10.0)	
**Diet drinks, n (%)**							
Never	4677	(66.1)	5827	(58.0)	993	(58.2)	
< 1 time/week	1341	(18.9)	2647	(26.3)	438	(25.7)	<0.001
1-6 times/week	594	(8.4)	1150	(11.4)	184	(10.8)	
≥ 1 times/day	469	(6.6)	428	(4.3)	90	(5.3)	
**NNS in coffee, tea, or drinks, n (%)**							
Never	4468	(63.1)	5146	(51.2)	879	(51.6)	
Either	2335	(33.0)	3976	(39.6)	701	(41.1)	<0.001
Both	278	(3.9)	930	(9.3)	125	(7.3)	
**Major depression[†], n (%)**	1248	(17.6)	1619	(16.1)	350	(20.5)	<0.001
**Province of residence, n (%)**							
Nova Scotia	4706	(66.5)	6030	(60.0)	1009	(59.2)	
New Brunswick	1368	(19.3)	2642	(26.3)	433	(25.4)	<0.001
Newfoundland and Labrador	894	(12.6)	1176	(11.7)	226	(13.3)	
Prince Edward Island	113	(1.6)	204	(2.0)	37	(2.2)	
**Ethnicity, n (%)**							
White	5518	(77.9)	8836	(87.9)	1504	(88.2)	
Non-white	354	(5.0)	585	(5.8)	103	(6.0)	<0.001
DNK/PNA	1209	(17.1)	631	(6.3)	98	(5.8)	
**Education, n (%)**							
Less than high school	1227	(17.3)	1813	(18.0)	352	(20.7)	
College level	2594	(36.6)	4006	(39.9)	686	(40.2)	<0.001
University level or higher	2430	(34.3)	4199	(41.8)	657	(38.5)	
DNK/PNA	830	(11.7)	34	(0.3)	10	(0.6)	
**Smoking status, n (%)**							
Never smoker	3816	(53.9)	4718	(46.9)	592	(34.7)	

Former smoker	1999	(28.2)	4296	(42.7)	750	(44.0)	<0.001
Current smoker	403	(5.7)	967	(9.6)	353	(20.7)	
DNK/PNA	863	(12.2)	71	(0.7)	10	(0.6)	
**Alcohol use, n (%)**							
Abstainer	1039	(14.7)	727	(7.2)	175	(10.3)	
Occasional drinker	3068	(43.3)	3695	(36.8)	658	(38.6)	
Regular drinker	1546	(21.8)	3589	(35.7)	525	(30.8)	<0.001
Habitual drinker	584	(8.3)	1989	(19.8)	335	(19.7)	
DNK/PNA	844	(11.9)	52	(0.5)	12	(0.7)	
**Cardiovascular disease‡, n (%)**	235	(3.3)	286	(2.9)	65	(3.8)	0.046
**Diabetes mellitus§, n (%)**	600	(8.5)	697	(6.9)	132	(7.7)	<0.001
**Body mass index, kg/m²**	28.5	(6.0)	28.2	(5.3)	29.0	(5.5)	<0.001
**Total physical activity, MET-min/week**	2079	(792, 4638)	2220	(904, 4843)	2656	(960, 5838)	<0.001
**Healthy eating index**	34.3	(14.9)	40.0	(7.9)	38.9	(8.2)	<0.001

DNK/PNA = do not know/prefer not to answer; NNS = non-nutritive sweetener.

*Data are means (standard deviation), number of participants (percentage), or median (interquartile range). Percentages may not total 100 due to rounding. The numbers of participants vary slightly among different variables.

†Defined as a PHQ-9 score ≥ 10 and/or current use of antidepressant.

‡Self-reported coronary heart disease and/or stroke.

§Self-reported diabetes mellitus.

Supplemental Table 2. Characteristics of participants according to non-nutritive sweeteners use*

Characteristics	NNS use: Never (n = 10493)		NNS use: Either|| (n = 7012)		NNS use: Both¶ (n = 1333)		*P* value
**Age, yr**	53.0	(9.1)	52.9	(8.9)	52.1	(9.2)	0.002
**Sex, n (%)**							
Men	3416	(32.6)	2072	(29.6)	366	(27.5)	<0.001
Women	7077	(67.4)	4940	(70.5)	967	(72.5)	
**Regular coffee, n (%)**							
Never	4963	(47.3)	2797	(39.9)	400	(30.0)	
> 0-< 4 cups/day	4776	(45.5)	3629	(51.8)	835	(62.6)	<0.001
≥ 4 cups/day	754	(7.2)	586	(8.4)	98	(7.4)	
**Decaffeinated coffee, n (%)**							
Never	9639	(91.9)	6213	(88.6)	1152	(86.4)	
> 0- < 2 cups/day	524	(5.0)	473	(6.8)	100	(7.5)	<0.001
≥ 2 cups/day	330	(3.1)	326	(4.7)	81	(6.1)	
**Total coffee, n (%)**							
Never	4468	(42.6)	2335	(33.3)	278	(20.9)	
> 0-< 4 cups/day	5146	(49.0)	3976	(56.7)	930	(69.8)	<0.001
≥ 4 cups/day	879	(8.4)	701	(10.0)	125	(9.4)	
**Major depression†, n (%)**	1633	(15.6)	1271	(18.1)	313	(23.5)	<0.001
**Province of residence, n (%)**							
Nova Scotia	6546	(62.4)	4400	(62.8)	799	(59.9)	
New Brunswick	2586	(24.7)	1578	(22.5)	279	(20.9)	<0.001
Newfoundland and Labrador	1150	(11.0)	904	(12.9)	242	(18.2)	
Prince Edward Island	211	(2.0)	130	(1.9)	13	(1.0)	
**Ethnicity, n (%)**							
White	8513	(81.1)	6199	(88.4)	1146	(86.0)	
Non-white	554	(5.3)	392	(5.6)	96	(7.2)	<0.001
DNK/PNA	1426	(13.6)	421	(6.0)	91	(6.8)	
**Education, n (%)**							
Less than high school	1830	(17.4)	1287	(18.4)	275	(20.6)	
College level	3847	(36.7)	2849	(40.6)	590	(44.3)	<0.001
University level or higher	3973	(37.9)	2851	(40.7)	462	(34.7)	
DNK/PNA	843	(8.0)	25	(0.4)	6	(0.5)	
**Smoking status, n (%)**							
Never smoker	5041	(48.0)	3516	(50.1)	569	(42.7)	
Former smoker	3632	(34.6)	2815	(40.2)	598	(44.9)	<0.001

Current smoker	940	(9.0)	630	(9.0)	153	(11.5)	
DNK/PNA	880	(8.4)	51	(0.7)	13	(1.0)	
**Alcohol use, n (%)**							
Abstainer	1073	(10.2)	726	(10.4)	142	(10.7)	
Occasional drinker	3806	(36.3)	2966	(42.3)	649	(48.7)	
Regular drinker	2975	(28.4)	2296	(32.7)	389	(29.2)	<0.001
Habitual drinker	1780	(17.0)	984	(14.0)	144	(10.8)	
DNK/PNA	859	(8.2)	40	(0.6)	9	(0.7)	
**Cardiovascular disease‡, n (%)**	299	(2.9)	233	(3.3)	54	(4.1)	0.026
**Diabetes mellitus§, n (%)**	569	(5.4)	640	(9.1)	220	(16.5)	<0.001
**Body mass index, kg/m^2**	27.6	(5.3)	29.2	(5.7)	30.3	(6.1)	<0.001
**Total physical activity, MET-min/week**	2239	(891, 4989)	2151	(843, 4579)	2306	(815, 5357)	0.002
**Healthy eating index**	36.2	(13.4)	39.6	(7.9)	39.8	(7.9)	<0.001

DNK/PNA = do not know/prefer not to answer; NNS = non-nutritive sweetener.

*Data are means (standard deviation), number of participants (percentage), or median (interquartile range). Percentages may not total 100 due to rounding. The numbers of participants vary slightly among different variables.

†Defined as a PHQ-9 score ≥ 10 and/or current use of antidepressant.

‡Self-reported coronary heart disease and/or stroke.

§Self-reported diabetes mellitus.

||Either added non-nutritive sweetener in coffee or tea or drank diet drinks.

¶Both added non-nutritive sweetener in coffee or tea and drank diet drinks.
